# Origin of photoinduced DC current and two-level population dynamics in a single molecule

**DOI:** 10.1126/sciadv.adk9211

**Published:** 2024-01-31

**Authors:** Jiang Yao, Youngwook Park, Wenlu Shi, Siyu Chen, W. Ho

**Affiliations:** ^1^ Department of Physics and Astronomy, University of California, Irvine, Irvine, CA 92697-4575, USA.; ^2^ Department of Chemistry, University of California, Irvine, Irvine, CA 92697-2025, USA.

## Abstract

Studying the photoinduced changes of materials with atomic-scale spatial resolution can provide a fundamental understanding of light-matter interaction. A long-standing impediment has been the detrimental thermal effects on the stability of the tunneling gap from intensity-modulated laser irradiation of the scanning tunneling microscope junction. Photoinduced DC current transduces photons to an electric current and is widely applied in optoelectronics as switches and signal transmission. Our results revealed the origin of the light-induced DC current and related it to the two-level population dynamics and related nonlinearity in the conductance of a single molecule. Here, we compensated for the near-visible laser-induced thermal effects to demonstrate photoinduced DC current spectroscopy and microscopy and to observe the persistent photoconductivity of a two-level pyrrolidine molecule. The methodology can be generally applied to the coupling of light to scan probes to investigate light-matter interactions at the atomic scale.

## Teaser

Solving thermal instability in laser irradiation of scanning tunneling microscope measures current in single-molecule dynamics.

## INTRODUCTION

Photoinduced DC current is the DC electrical response to light and has been widely observed in macroscopic nonlinear systems (*1*–*5*) and metallic tunneling junctions with near-field plasmon enhancement (*6*, *7*). In addition, single-molecule spectroscopy based on photoinduced DC current measurements in the terahertz and RF frequencies can reach ultrahigh energy and spatial resolution when combined with scanning tunneling microscopy (STM) (*8*–*16*). In these measurements, the intensity modulation combined with lock-in detection was applied to extract the weak photoinduced DC current. However, the intensity modulation of light from mid-infrared (IR) to ultraviolet frequencies introduces a long-standing problem of thermal effects that causes instabilities in the STM tunneling gap (*6*, *17*). A detailed study also directly showed that, with a chopped laser, the thermally induced mechanical oscillation was the major contribution to the optically induced electrical signal of a tip-sample tunneling junction (*18*). To date, two studies circumvented the “thermal problem” and reported photoinduced DC current in STM coupled to IR and visible radiation by using either high-frequency modulation (*19*) or weak laser power (*20*). However, high modulation frequency requires high laser power (>100 mW) because of the large attenuation at high frequencies of the high-gain preamplifier used in STM measurement. Such high laser power can destabilize the tunneling junction and desorb target molecules. Using weak power (<100 μW) limits studies to systems with large photocurrent response and extinction ratio, possibly for electronic states. A universal solution to the “thermal problem” would broaden the use of scan probes to implement photoinduced DC current spectromicroscopy at the atomic scale.

Persistent photoconductivity (PPC) is a phenomenon associated with the persistence of the photoinduced conductance change after the removal of light irradiation (*21*–*24*). Such effect has been widely observed in semiconductors and applied to photodetectors (*25*, *26*), memory devices (*27*, *28*), radiation detection (*29*, *30*), and solar cells (*31*–*33*). The recent observation of the PPC effect in monolayer transition metal dichalcogenides material could reduce the thickness of these photoelectronics (*34*–*36*). The miniaturization in all dimensions may rely on single molecules performing as key elements. However, the PPC effect of a single molecule has not been found. The PPC effect of the semiconducting system can all be attributed to the long lifetime of photoexcited carriers (*21*–*36*). Because of the absence of periodic structure, single molecules often lack energy bands, and the concept of carriers in single molecules is ill-defined. Therefore, the observation of PPC in single molecules requires the invention of a new mechanism.

## RESULTS

**Fig. 1.**
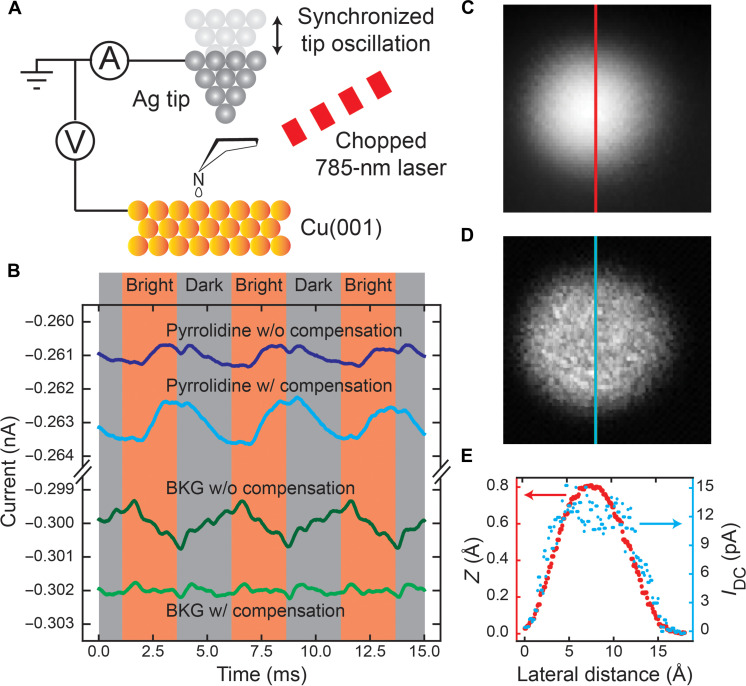
Atomic-scale photoinduced DC current measurement. (**A**) Schematic diagram of STM photoinduced DC current measurement. The synchronized tip oscillation was used to compensate for the oscillatory thermal expansion of the tunneling gap induced by laser chopping. (See the Supplementary Materials for details of the compensation procedures.) (**B**) Current traces taken over a pyrrolidine molecule and over the Cu(001) background with (w/) and without (w/o) Z-compensation. Before measurement, the feedback loop was first opened at −250 mV/0.75 nA, followed by ramping the sample bias to −100 mV. A constant 3.15-mW CW laser was first chopped at 201 Hz and then focused into the STM junction, and a 12.8-pm amplitude tip oscillation with a phase shift of 74.2° was used for the Z-compensation. All traces in (B) were averaged 20,000 passes. (**C**) Topography and (**D**) photoinduced DC current of pyrrolidine simultaneously imaged. (See the Supplementary Materials for measurement and image processing details.) The photoinduced DC current was measured at a constant conductance set by −250 mV/0.75 nA, followed by ramping the bias to −100 mV. The amplitudes of laser power modulation and Z-oscillation were 0.48 mW and 3.7 pm, respectively. The phase shift for Z-oscillation was 86.0°. We chopped the laser at 327 Hz for all photoinduced DC current measured with lock-in amplifier unless specified otherwise. (**E**) Vertical line cut of (C) (red) and (D) (blue) with red and blue arrows pointing to the corresponding *y* axis. Data in (C) to (E) were obtained with a different tip than the one for data in (B).

Here, we demonstrated the Z-compensation technique to counter the laser modulation-induced “thermal problem” of the STM junction instability. We applied a synchronized tip oscillation perpendicular to the sample surface to compensate for the thermally induced tunneling gap oscillation due to chopping of the continuous wave (CW) laser light (Fig. 1A). The laser heating did not require compensation in directions parallel to the surface. The tip oscillation amplitude was tuned to be the same as the tunneling gap oscillation but with a 180° phase difference to nullify the thermal effect (see the Supplementary Materials for the compensation details). The best compensation conditions were achieved by minimizing the tunneling current oscillation amplitude over the substrate surface (background) (Fig. 1B). Such conditions were then used to measure photoinduced DC current over the molecule.

To demonstrate the effectivity of the Z-compensation method, we measured 785-nm CW light-induced DC current over a single pyrrolidine molecule adsorbed on the Cu(001) surface at 6 K with a home-built STM. We observed clear tunneling current oscillations in the total DC current (STM tunneling current plus photoinduced DC current) with laser intensity modulation and proper Z-compensation (Fig. 1B). Theoretically, the chopping method with a square wave modulates the laser intensity and introduces a mechanical oscillation of the tunneling gap with higher harmonic components. However, we found that higher harmonic compensation is not necessary for the measurement because the thermal expansion of the tunnel junction slowly responded to the temperature change. Such a slow response acted as a low pass filter and attenuated all higher harmonic temperature oscillations (see fig. S6D). In addition, the slow response of the photoinduced electric current signal also proved that the thermally induced mechanical oscillation was the major contribution to the background signal. Other photo-assisted electrical transport mechanisms, such as hot electron photocurrent, are expected to have a much faster response.

The topography of the molecule is shown in Fig. 1C. The weak photoinduced DC current signal was extracted from the total current with a lock-in amplifier and a two-dimensional mapping of the photoinduced DC current signal was obtained (Fig. 1D). These images exhibit submolecular contrast (Fig. 1, C to E). The topography shows round protrusion, while the photoinduced DC current image exhibits a donut shape with a small dip at the central plateau. We further investigated the origin and relation of the photoinduced DC current to the molecular dynamic response by comparing the results of the experiment to modeling based on single-molecule two-state switching. The line shape of photoinduced DC current spectra also provides direct evidence of the single-molecule PPC effect, which is driven by a novel mechanism of photo-assisted molecular conformational switching.

**Fig. 2.**
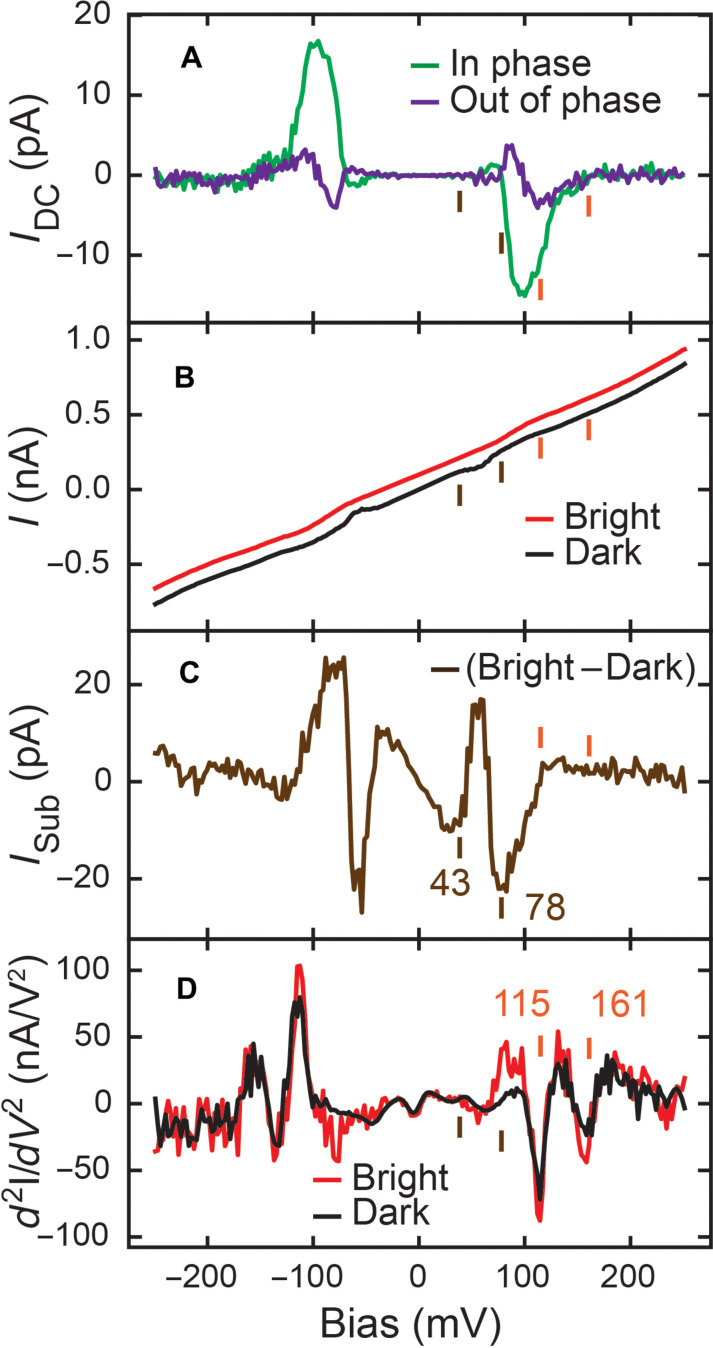
*I*_DC_(*V*) and *I*_sub_(*V*) of a pyrrolidine molecule on Cu(001). (**A**) *I*_DC_(*V*) of pyrrolidine with 0.97-mW modulated laser power and 8.1-pm Z-oscillation amplitude with a phase shift of 97.7°. (**B**) *I*(*V*) of pyrrolidine with constant 0.97-mW unmodulated laser illumination (bright) and without laser illumination (dark). There was a 300-ms wait time before measuring each data point. The bright current is shifted up by 0.1 nA for clarity. (**C**) The result of bright current subtracting dark current in (B). (**D**) IETS (*40*) of pyrrolidine under the same dark and bright conditions as (B). We used 7 mV_rms_ at 397.48-Hz bias modulation for IETS measurement. (A), (B), and (D) were measured over the center of the pyrrolidine molecule, with a constant tunneling gap feedback set point −250 mV/0.75 nA. The short orange and brown lines indicate the dip positions in (B) and (D).

The laser chopping method with lock-in detection measures photoinduced DC current, *I*_DC_, which should reflect the current difference between constant laser irradiation (bright) and without laser irradiation (dark). However, the measured *I*_DC_ as a function of sample bias [*I*_DC_(*V*); Fig. 2A] differs from the bright *I*(*V*) subtracting the dark *I*(*V*) [*I*_sub_(*V*) = *I*_bright_(*V*) − *I*_dark_(*V*)], in Fig. 2 (B and C). On the other hand, both the *I*_DC_(*V*) (Fig. 2A) and *I*_sub_(*V*) (Fig. 2C) differ from the measured $\frac{d^{2}I}{{\mathrm{dV}}^{2}}$ [Fig. 2D, from the conventional inelastic electron tunneling spectroscopy (IETS)]. Furthermore, we did not observe *I*_DC_ signal over a carbon monoxide (CO) molecule adsorbed on Cu(001), which exhibited a strong $\frac{d^{2}I}{{\mathrm{dV}}^{2}}$ (IETS) signal below 50 mV (see fig. S9). Both phenomena suggest that the optical field plays a minor role in the measured pyrrolidine photoinduced DC current (*6*–*9*, *37*). Therefore, to understand the observed photoinduced DC current, we need to answer two questions: (i) Why does the *I*_DC_(*V*) deviate from the *I*_sub_(*V*)? (ii) What is the origin of the *I*_DC_ and *I*_sub_? The out-of-phase signal (Fig. 2A) indicates that the signal phase of *I*_DC_ varies as the bias changes. Such phase variation implies varying system dynamic responses at different biases, requiring an understanding of the dynamics of the pyrrolidine molecule.

**Fig. 3.**
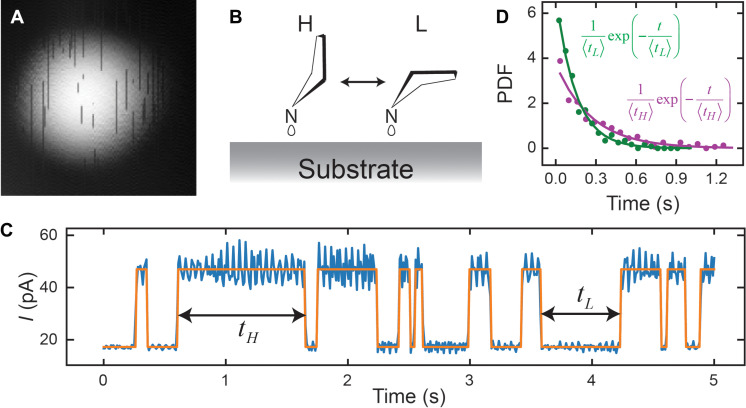
Switching dynamics of pyrrolidine on Cu(001). (**A**) Constant current topography of pyrrolidine at feedback set point −30 mV/100 pA. (**B**) Schematic of pyrrolidine conformational switching between H and L states. (**C**) Time trace of the tunneling current over the topographic center of pyrrolidine. High and low currents indicate H and L states. Before measurement, a feedback loop was opened after setting the tunneling gap at −250 mV/100 pA, followed by ramping the bias to 73 mV. (**D**) The probability density function of residence time for H (purple) and L (green) states. The statistical data were generated from two current traces of 100-s duration, measured with the same conditions as (C). The solid curves are Poisson distributions calculated from the formula displayed in the same color. 〈*t_H_*〉 = 267 ms and 〈*t_L_*〉 = 149 ms are the mean values of *t_H_* and *t_L_*.

To understand the origin of the *I*_DC_ and *I*_sub_, we investigate the behavior of pyrrolidine molecule at larger tip-molecule separation. The telegraphic noise in low-bias topography (Fig. 3A) supports the switching of pyrrolidine between two conformational states (H and L), the single-molecule states, as depicted in Fig. 3B (*38*–*41*). Current traces monitor such switching process and show two current levels (Fig. 3C). The high and low current levels correspond to H and L states (Fig. 3B). The state residence time, *t*_*L*/*H*_, follows Poisson distribution, which appears as an exponential decay in its probability density function (Fig. 3D). The decay rate defines the corresponding transition rate, *W*_*L*/*H*→*H*/*L*_, which equals the inverse of the mean value of *t*_*L*/*H*_ (*42*). Such exponential decay suggests that the escaping rate out of the corresponding state equals *n*_*L*/*H*_*W*_*L*/*H*→*H*/*L*_ (*43*, *44*), where *n*_*L*/*H*_ is the probability of molecule in the L (H) state. Therefore, combined with probability conservation, a differential equation (Eq. 1) models the response of pyrrolidine switching dynamics.

$$\frac{\partial }{\partial t}\left[\frac{n_{H}\left(V,t\right)}{n_{L}\left(V,t\right)}\right]=\left[\begin{array}{cc} -W_{H\to L}\left(V,t\right) & W_{L\to H}\left(V,t\right)\\ W_{H\to L}\left(V,t\right) & -W_{L\to H}\left(V,t\right) \end{array}\right]\left[\frac{n_{H}\left(V,t\right)}{n_{L}\left(V,t\right)}\right]$$ (1)

**Fig. 4.**
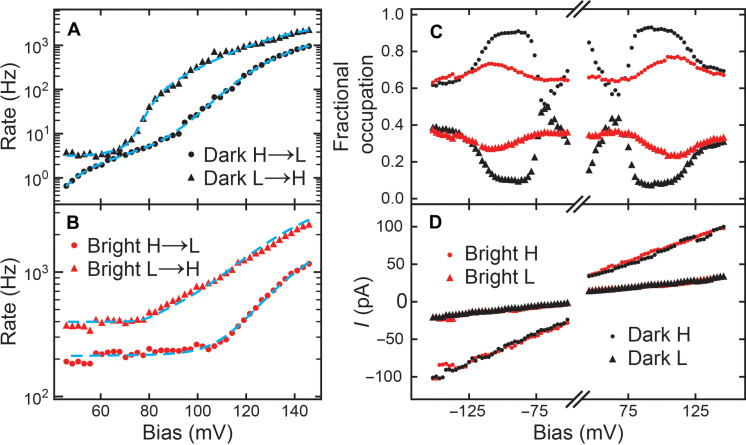
Bias-dependent switching dynamics of pyrrolidine on Cu(001). (**A** and **B**) Transition rates as a function of sample bias under dark (black) and bright (irradiation with unmodulated, constant 0.97-mW laser, red) conditions. The negative bias data are not shown because of limited space and are presented in the Supplementary Materials. The blue dashed lines in (A) fit the data based on the inelastic tunneling electron switching model. *H* → *L* rate (dot) was fitted with out-of-plane ring mode (43.3 mV) and ring breathing mode (120.4 mV). *L* → *H* rate (triangle) was fitted with in-plane ring mode (77.9 mV) and ring breathing mode (111.0 mV). (See the Supplementary Materials for the fitting details.) The mode softening between two different conformations could contribute to the different excitation energies for the same mode. The blue dashed lines in (B) are shifted up by a constant from the corresponding lines in (A): *H* → *L* by 200 Hz and *L* → *H* by 380 Hz. (**C**) Occupation percentage as a function of bias for the H (dot) and L (triangle) states of pyrrolidine under bright and dark conditions. (**D**) State-resolved tunneling current under bright and dark conditions measured over the topographic center of pyrrolidine. All statistics displayed in (A) to (D) were extracted from multiple long current traces at each bias with the same tunneling gap set as Fig. 3C.

To solve for *n*_*H*/*L*_ in Eq. 1, we measured *W*_*L*/*H*→*H*/*L*_ under dark and bright conditions. The inelastic tunneling electron-assisted switching dominates the dark transition rates (*42*, *45*–*47*), *W*_*L*/*H*→*H*/*L*,dark_, and the fitted results based on this model agree with the measurement (Fig. 4A). By adding 395.5 and 211.4 Hz to the fitted dark transition rates of *L* → *H* and *H* → *L* processes, we produce the solid curves in Fig. 4B, which agrees with the experimentally measured bright transition rates, *W*_*L*/*H*→*H*/*L*,bright_. Such good fitting indicates voltage-independent light assistance to the pyrrolidine switching process. We also find such light-assisted switching rates to be tip-dependent, which indicates the possible involvement of tip enhanced plasmon effect (*48*, *49*). For time-invariant transition rates *W*_*L*/*H*→*H*/*L*_, the steady-state solution of Eq. 1 gives the percentage of occupation in the H and L states

$$n_{H/L,\infty }\left(V\right)=\frac{W_{L/H\to H/L}\left(V\right)}{W_{L\to H}\left(V\right)+W_{H\to L}\left(V\right)}$$ (2)

The difference between bright and dark transition rates leads to different *n*_*H*/*L*,∞_(*V*) (Fig. 4C).

On the other hand, linear relations of state-resolved *I*(*V*) (Fig. 4D) suggest constant H and L conductances (σ*_H_* and σ*_L_*) which are insensitive to light irradiation. We extract the σ*_H_* and σ*_L_* from the slope of the corresponding state *I*-*V* curve in Fig. 4D, which are 0.658 and 0.174 nS. Therefore, the total tunneling current can be written as follows

$$I\left(V\right)=\left[n_{H}\left(V\right)\sigma_{H}+n_{L}\left(V\right)\sigma_{L}\right]V=\left[n_{H}\left(V\right)∆\sigma +\sigma_{L}\right]V$$ (3)

∆σ is the conductance difference between H and L states, σ*_H_* − σ*_L_*. The final expression in Eq. 3 makes use of the normalization condition of state probability, *n_H_* + *n_L_* = 1. Because the laser radiation can induce the change in *n_H_*(*V*) (Fig. 4C) and the change in *n_H_*(*V*) contributes to the current change (Eq. 3), we deduce that the photoinduced DC current originates from a light-induced change in population *n_H_*(*V*).

**Fig. 5.**
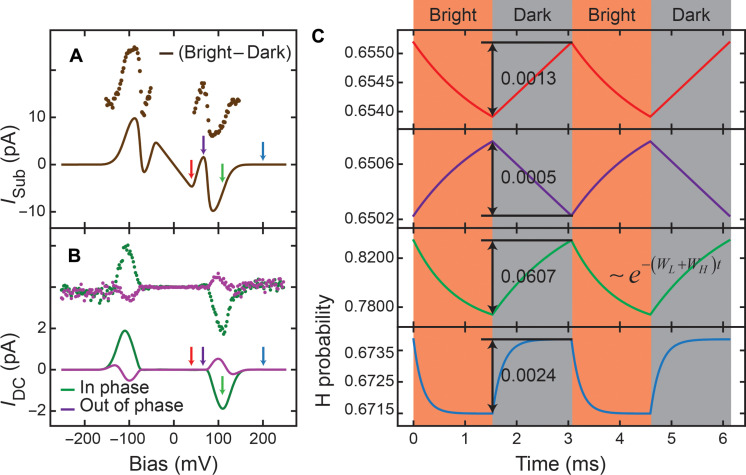
Origin of photoinduced DC current of pyrrolidine on Cu(001). (**A**) The simulated (solid lines) and measured (dots) results of current under bright conditions subtracting current under dark conditions. The bright and dark currents were measured by averaging the same current traces used for statistics in Fig. 4 (A and B). (**B**) The simulated (solid lines) and measured (dots) *I*_DC_(*V*). The measured *I*_DC_(*V*) were acquired with modulated 0.97-mW laser power, 8.1-pm Z-oscillation amplitude with a phase shift of 81.8° and a constant tunneling gap determined by feedback set point of −250 mV/0.75 nA. The experimental data in (A) and (B) are shifted up by 15 and 4 pA for clarity. (**C**) Simulated time evolution of pyrrolidine H state probability at different sample biases: 40 mV (red), 67 mV (purple), 109 mV (green), and 200 mV (blue). The four biases are also indicated in (A) and (B) with corresponding color arrows. Note the different vertical scales.

To verify our deduction, we further calculate the *I*_sub_ (*V*) by incorporating the fitted transition rate data (green dashed curve in Fig. 4, A and B) into Eqs. 2 and 3. Figure 5A shows the excellent agreement between the calculated *I*_sub_(*V*) (solid line) and measurement (dots). Next, we focus on revealing the reason for the deviation of *I*_DC_ (*V*) from *I*_sub_ (*V*). One major difference between *I*_DC_ (*V*) and *I*_sub_ (*V*) arises from the dynamics of the pyrrolidine conformational change. Here, *I*_sub_ (*V*) is the difference in the DC current between two steady-state conditions, bright and dark, while *I*_DC_ (*V*) measures the time-dependent changes in the DC current induced by the laser intensity modulation. Laser intensity modulation adds time dependence to the *W*_*L*/*H*→*H*/*L*_ expression

$$W_{L/H\to H/L}\left(V,t\right)=W_{L/H\to H/L,\text{dark}}\left(V\right)+W_{L/H\to H/L,\text{ph}}h\left(t\right)$$ (4)

The square wave function, *h*(*t*), oscillates between 0 and 1 at laser power modulation frequency, $\frac{\omega }{2\pi }$ . *W*_*L*/*H*→*H*/*L*,ph_ is the photon contribution to the transition rates and equals *W*_*L*/*H*→*H*/*L*,bright_ − *W*_*L*/*H*→*H*/*L*,dark_. The origin of *W*_*L*/*H*→*H*/*L*,ph_ likely involves the decay of localized surface plasmons to generate hot electrons that excite pyrrolidine vibrations directly between the two levels in a double-well potential to facilitate the switching process (see the Supplementary Materials).

Adopting *W*_*L*/*H*→*H*/*L*_(*V*, *t*) form in Eq. 4 and using the fitted dark and bright transition rates in Fig. 4 (A and D), we numerically calculate *n_H_*(*V*, *t*) using Eq. 1 and plug it into Eq. 3 to obtain the time-dependent changes in the tunneling current *I*(*V*, *t*). The lock-in measurement extracts the first harmonic component of *I*(*V*, *t*) at the laser power modulation frequency, *I*_DC_(*V*). The calculated *I*_DC_(*V*) using the above method agrees with the experimental measurement (Fig. 5B). Therefore, *I*_DC_(*V*) reflects the dynamic *n_H_* response to a square wave chopped laser illumination. Both *I*_DC_ and *I*_sub_ originate from the *n_H_* response to the laser radiation, while their spectral shape exhibits different features. To understand such differences, we further calculate the time evolution of *n_H_* at four different biases (Fig. 5C).

At low biases [40 mV (red) and 67 mV (purple)], *W*_*L*/*H*→*H*/*L*,ph_ is notably larger than *W*_*L*/*H*→*H*/*L*,dark_. The bright and dark *n*_*H*,∞_ will differ from each other as long as $\frac{W_{L\to H,\text{dark}}}{W_{H\to L,\text{dark}}}\neq \frac{W_{L\to H,\mathrm{ph}}}{W_{H\to L,\mathrm{ph}}}$ (Eq. 2). Finite values for *I*_sub_ arise from this difference (Eq. 3). However, *I*_DC_ reflects *n_H_* variation under the irradiation of chopped laser in the tunneling junction. Upon chopping the laser for intensity modulation, *n_H_* starts to exponentially approach *n*_*H*,∞_ at a rate of *W*_*H*→*L*_ + *W*_*L*→*H*_ (Fig. 5C, see also equation 7 in the Supplementary Materials). However, before the next laser intensity change, *n_H_* only has limited time to evolve during half of the modulation period. If this half period is much longer than the exponential decay time constant, 1/(*W*_*H*→*L*_ + *W*_*L*→*H*_)], *n_H_* can reach its equilibrium value. But the half modulation period that we used is 1.53 ms, thus *n_H_* can only have limited variation and correspondingly near-zero *I*_DC_. We describe the signal attenuation due to the slow system response as the dynamical attenuation. We can expect this if the modulation frequency is sufficiently low or the molecule switches at a high rate such as at high bias, *I*_DC_ approaches *I*_sub_.

At a high bias [200 mV (blue)], the inelastic tunneling electron contribution to the transition rates is much higher than the photon contribution, which yields *W*_*L*/*H*→*H*/*L*,dark_ ≈ *W*_*L*/*H*→*H*/*L*,bright_ and correspondingly negligible *n_H_* difference between bright and dark conditions (Eq. 2). Therefore, both *I*_DC_ and *I*_sub_ are close to zero.

At an intermediate bias [109 mV (green)], *W*_*L*/*H*→*H*/*L*,ph_ is comparable to *W*_*L*/*H*→*H*/*L*,dark_, which can introduce a difference in *n*_*H*,∞_ between dark and bright. The dynamic attenuation effect is still not too severe to entirely suppress the signal. Therefore, the *I*_DC_ overall appears as a peak. In addition, if the laser chopping frequency is low enough, *n_H_* can reach the corresponding *n*_*H*,∞_ in each half of the modulation cycle and its time evolution approaches square wave instead of triangular function, which results in *I*_DC_ ≈ *I*_sub_. Therefore, *I*_DC_(*V*) becomes *I*_sub_(*V*) at a low chopping frequency, equivalent to a low pass filtering effect. In practice, we observe decay in *I*_DC_ as the chopping frequency increases (see fig. S6C).

From the above analysis, we can conclude that the spectral difference between *I*_DC_ and *I*_sub_ originates from the slow *n_H_* response to laser radiation. In this process, the tunneling conductance determined by *n_H_* (Eq. 3) persists a while after switching on and off the light, which reflects the single-molecule PPC effect of pyrrolidine. On the other hand, the *I*_DC_ measurements exhibit strong dependences on the tunneling current (see fig. S7) and sample bias (Fig. 2A). These dependencies suggest that the single-molecule PPC effect can be controlled by the electron current and energy.

Any laser characteristics that affect the transition rates can potentially change *I*_DC_ by changing *n*_*H*,∞_ and the dynamic response of the molecule (see the Supplementary Materials). For example, change in laser power and polarization can affect the transition rate and lead to change in *I*_DC_(*V*) (see fig. S8). In addition, the response of the molecular transition rate exhibits laser wavelength dependence (*50*) and the time delay between two ultrafast pump-probe laser pulses (*38*). Therefore, we expect *I*_DC_ to vary with photon energy and pump-probe delay time, which could serve as spectroscopic tools to characterize single-molecule coherence and dynamics in the frequency and time domains.

## DISCUSSION

In summary, we implement a Z-compensation technique to suppress laser-induced thermal instability of the STM junction and measure the dynamic photoinduced DC current of a single pyrrolidine molecule. Such photoinduced DC current originates from changes in the two-state occupation induced by photon-assisted conformational switching. The inverse of the total switching rate exhibited by the molecule determines the response time of the state occupation probability. Such finite single-molecule response leads to the different spectral line shapes between photoinduced DC current arising from the intensity modulation of the fixed frequency laser and the subtraction of the steady-state DC currents for bright and dark. Inelastic tunneling electrons and photons simultaneously induce the two-state switching dynamics for a single molecule. Photoinduced DC current is expected in other photo-switching molecular systems. The Z-compensation technique solves the long-standing thermal problem caused by laser irradiation of the scan probe junction and enables the study of single-molecule dynamics and spectromicroscopy based on the measurement of photoinduced DC current at optical frequencies.

## MATERIALS AND METHODS

The experiments were performed using a home-built ultrahigh vacuum STM operating at 6 K. The microscope was cooled by a continuous flow liquid helium cryostat (ARS, Helitran LT3B) with helium supplied by a flexible transfer line connected to a 100-liter dewar. The tips used in the experiments were prepared by electrochemical etching of a 0.051-cm diameter silver wire and subsequent in-vacuum cycles of sputtering with Ne^+^ (8 × 10^−5^ torr) and annealing. The Cu(001) surface was cleaned by cycles of Ne^+^ sputtering, followed by annealing to around 800 K. The pyrrolidine chemical (98% purity, Spectrum Chemical) was purified by around 50 freeze-pump-thaw cycles. The molecules at room temperature were evaporated onto Cu(001) surface held at 6 K.

A tunable CW diode laser (DFB pro, TOPTICA) was used to generate 785-nm wavelength light. The laser beam was directed into an acoustic optical modulator to control and chop the laser intensity (fig. S1). The chopped laser beam was focused into the tunneling junction with a 7.6-cm focal length fused silica lens.

## Supplementary Materials

### This PDF file includes:

Supplementary Text Figs. S1 to S10 Tables S1 and S2 References
