# Supplementary material for: Origin of photoinduced DC current and two-level population dynamics in a single molecule
Source: Sci Adv. 2024 Jan 31;10(5):eadk9211. doi: 10.1126/sciadv.adk9211 (PMC10830102; doi:10.1126/sciadv.adk9211)
Supplement: Supplementary file 1 — Supplementary Text Figs. S1 to S10 Tables S1 and S2 References [file sciadv.adk9211_sm.pdf]

Science Advances
AAAS

Supplementary Materials for

Origin of photoinduced DC current and two-level population dynamics in a single molecule

Jiang Yao *et al.*

Corresponding author: W. Ho, wilsonho@uci.edu

Sci. Adv. **10**, eadk9211 (2024)
DOI: 10.1126/sciadv.adk9211

The PDF file includes:

Supplementary Text
Figs. S1 to S10
Tables S1 and S2
References

Thermal Z-Compensation

Shining a square-wave chopped laser into the STM junction caused thermal oscillation of the tunneling gap because of the uneven thermal expansion of tip and sample. The thermally induced tip motion parallel to the surface was not observed. Such thermal oscillation in the Z-direction was synchronized with a phase shift to the laser intensity modulation. Therefore, the thermal effect could be suppressed by adding a synchronized oscillation of the tip to compensate for the thermally induced tunneling gap oscillation. The added compensation to the tunneling gap thermal oscillation needed to have the same amplitude as the thermal oscillation but with 180-degree phase difference to nullify the thermal effect.

Theoretically, the square-wave chopped laser would introduce a square-wave-like thermal oscillation, thus requiring a complicated tip oscillation waveform with many harmonics for the compensation. However, in practice, the thermal expansion responded slowly to the laser intensity modulation, thus all the higher harmonics were suppressed, which resulted in a near-sinusoidal thermal oscillation. After proper Z-compensation with a sinusoidal tip oscillation, we monitored the fast Fourier transform (FFT) of the tunneling current with tip parked over the metal substrate in constant height mode. We did not observe any higher harmonic signals in the tunneling current after sinusoidal compensation of the chopped laser intensity. During this process, the tunneling gap was kept small with 50 mV/2 nA set point. A sinusoidal tip oscillation was found to be sufficient to compensate for the laser induced thermal effects. The optimal compensation signal measured the thermal oscillation amplitude, which decayed as laser chopping frequency increased (Fig. S4D). Such phenomena also verified the slow response of thermal expansion.

Figure S1A shows the schematic diagram for compensating thermally induced tunneling gap oscillation. The tip oscillation compensation was generated by feeding a synchronized voltage oscillation (Z-oscillation) to the tip piezo. An arbitrary function generator (AFG31022 Tektronix) provided both the Z-oscillation and the laser intensity modulation signals and guaranteed the synchronization between these two signals. We measured tunneling gap oscillation by monitoring the tunneling current oscillation with a current amplifier DL1211 and a lock-in amplifier DSP 7265, while the tip was parked over the Cu(001) background at a constant height by turning off the feedback. The Z-oscillation signal amplitude and phase were varied until the oscillation signal in the current disappeared.

The sequential steps for performing the thermal compensation are described below:

1. Park the STM tip over Cu(001) background with the feedback on.
2. Irradiate square wave chopped laser into the STM junction and wait the order of a few minutes until sample temperature stabilizes.
3. Ramp bias and current, so that tip is close to surface (e.g., 50 mV/2 nA).
4. Turn feedback off.
5. Align lock-in amplifier reference phase with thermal oscillation induced tunneling current signal.
6. Turn on small amplitude Z-oscillation. (e.g., 73 pm amplitude).
7. Record the in-phase and out-of-phase signal with lock-in amplifier as a function of Z-oscillation phase (Fig. S1B).

8. Fit the out-of-phase signal as a function of Z-oscillation phase with a sine function to determine the phase, $\theta_{optimal}$, that Z-oscillation superpose destructively with thermally induced gap oscillation.
9. Set Z-oscillation phase to $\theta_{optimal}$.
10. Record the in-phase and out-of-phase signals with lock-in amplifier as a function of Z-oscillation amplitude.
11. Fit the in-phase signal as a function of Z-oscillation amplitude with a linear function to determine the optimal amplitude, $A_{optimal}$, that Z-oscillation eliminates the signal induced by thermal effect (Fig. S1C).
12. Set Z-oscillation amplitude to $A_{optimal}$.
13. Turn feedback on.
14. Ramp bias and current to the original conditions.

The thermal oscillation signal originated from the tip Z-oscillation followed approximately by $\frac{dI}{dZ}$. Since the tunneling current exponentially depended on Z, $\frac{dI}{dZ} \sim I$ (*6*). Thus, there appeared a tilted base line in uncompensated $I_{DC}(V)$ (Fig. S1D and Fig. S1E) because the *I(V)* curve typically was linear for metallic surfaces at low bias voltages. The signal of tip oscillation without laser irradiation showed a baseline with a slope of opposite sign (Fig. S1D and Fig. S1E). The compensated $I_{DC}(V)$ combined these two signals and resulted in a flat base line and preserved the molecular features (Fig. S1D and Fig. S1E).

Any changes in the thermal oscillation would affect the optimal compensation condition. Therefore, we repeated the compensation procedures when a change occurred in the laser alignment, power, or modulation frequency, or when the tip re-approached the surface and landed in a different area.

Photoinduced DC current Image Processing

To minimize the XY drift and to reduce the probability that the molecule hopped during imaging, we shortened the imaging time by reducing the image size to 64×64 pixels. Contrast was improved by averaging two photoinduced DC current images of 64×64 pixels, as shown in Fig. 1C, Fig. S2A, and Fig. S2B. First, we manually removed overloaded pixels and resized the photoinduced DC current image to 256×256 pixels with nearest neighbor algorithm. Second, we applied the pattern matching algorithm to the topography measured simultaneously (Fig. 2I and Fig. 2J) to locate the center of the molecule. Third, we put the two photoinduced DC current images on a canvas of 512×512 pixels with the center of the molecule positioned at the canvas center and with the two photoinduced DC current images occupying the same pixels on the canvas and averaged. Finally, we only include the pixels where the two photoinduced DC current images overlap. The results are shown in (Fig S2C). The out-of-phase image (Fig. S2G) was obtained by averaging Fig. S2E and Fig. S2F, following the same procedures. The line cuts across the in-phase image (Fig. S2D) and the topography (Fig. S2L) differ from each other, revealing the sub-molecular contrast resolved in the photoinduced DC current signal. The line cut (Fig. S2H) of the out-of-phase image is too noisy to draw any definitive conclusion.

Transition Rate Fitting

Inelastic tunneling electrons can assist single molecule switching through vibrational excitation and dominate the dark transition rates (*42, 45-47*), $W_{L/H\rightarrow H/L,dark}$:

$$W_{L/H\rightarrow H/L,dark}(V) = \sum_{\nu} K_{\nu} I_{IET,\nu}^{m_{\nu}}(V) + const_{L/H\rightarrow H/L} \quad (S1)$$

$\sum_{\nu} K_{\nu} I_{IET,\nu}^{m_{\nu}}(V)$ represents the inelastic electron induced transition rate, which is the linear sum of contributions from all vibrational modes involved in the inelastic tunneling process, where ν is the index for the vibrational modes. K_{ν}, $I_{IET,\nu}(V)$ and m_{ν} are the pre-factors, inelastic tunneling current, and multi-electron factor associated with the vibrational mode ν; $m_{\nu} = 1$ for single electron process. The term $const_{L/H\rightarrow H/L}$ represents potential contributions from nucleus tunneling and thermally induced switching. We used the following form for $I_{IET,\nu}(V)$ (*51*):

$$I_{IET,\nu}(V) = \frac{\hbar\Omega_{\nu} - eV}{e^{\left(\hbar\Omega_{\nu} - eV\right)/k_B T_{eff}} - 1} - \frac{\hbar\Omega_{\nu} + eV}{e^{\left(\hbar\Omega_{\nu} + eV\right)/k_B T_{eff}} - 1} \quad (S2)$$

The $W_{L/H\rightarrow H/L,dark}(V)$ data in Fig. 4A and Fig. S3A were fitted using Eq. S1 and S2 and the fitting parameters are shown in Table S1. The m_{ν} values were kept to 1 since the multi-electron effect was small under the measurement conditions of Fig. 4B and Fig. S3A, where the tunneling current was smaller than 100 pA.

The bright transition rates, $W_{L/H\rightarrow H/L,bright}(V)$, differ from $W_{L/H\rightarrow H/L,dark}(V)$ by fixed values (Fig. S3A). Such difference is attributed to the photon contribution, $W_{L/H\rightarrow H/L,ph}$, which is bias independent for pyrrolidine on Cu(001):

$$W_{L/H\rightarrow H/L,bright}(V) = W_{L/H\rightarrow H/L,dark}(V) + W_{L/H\rightarrow H/L,ph} \quad (S3)$$

The fitting results of $W_{L/H\rightarrow H/L,bright}(V)$ data shown in Fig. 4B and Fig. S3B were obtained by shifting the fitting result of $W_{L/H\rightarrow H/L,dark}(V)$ by constant values. The values of $W_{L/H\rightarrow H/L,ph}$ are listed in Table S2.

Photoinduced DC current Theoretical Derivation

The total probability for molecule occupying either H state or L state must equal to 1, because of the requirement of normalization.

$$n_H(V,t) + n_L(V,t) = 1 \quad (S4)$$

The escaping rate out of L/H state equals $n_{L/H}(V,t)W_{L/H\rightarrow H/L}(V,t)$. Because of the probability conservation:

$$\frac{\partial}{\partial t}\begin{pmatrix} n_H(V,t) \\ n_L(V,t) \end{pmatrix} = \begin{pmatrix} -W_{H\to L}(V,t) & W_{L\to H}(V,t) \\ W_{H\to L}(V,t) & -W_{L\to H}(V,t) \end{pmatrix}\begin{pmatrix} n_H(V,t) \\ n_L(V,t) \end{pmatrix} \quad (S5)$$

Plugging Eq. S4 into Eq. S5 results:

$$\frac{\partial n_H(V,t)}{\partial t} = -n_H(V,t)\big(W_{L\to H}(V,t) + W_{H\to L}(V,t)\big) + W_{L\to H}(V,t) \quad (S6)$$

If $W_{L\to H}$ and $W_{H\to L}$ are time-invariant, the solution to Eq. S6 is:

$$n_H(V,t) = \big(n_H(V,t_0) - n_{H,\infty}\big)e^{-(W_{L\to H}(V)+W_{H\to L}(V))(t-t_0)} + n_{H,\infty} \quad (S7)$$

$$n_{H,\infty} = \frac{W_{L\to H}(V)}{W_{L\to H}(V) + W_{H\to L}(V)} \quad (S8)$$

t_0 is the initial time. As $t_0 \to -\infty$, n_H approaches $n_{H,\infty}$, which gives the percentage occupation of H state in steady state dark and bright conditions.

With square-wave chopped laser, $W_{L\to H}$ and $W_{H\to L}$ are time-dependent and take the following form:

$$W_{L/H\to H/L}(V,t) = W_{L/H\to H/L,dark}(V) + W_{L/H\to H/L,ph}h(t) \quad (S9)$$

With the transition rates in Eq. S9, we didn't derive the analytic solution of Eq. S6 but solved it numerically (Fig. S4A to Fig. S4D). Since $W_{L/H\to H/L}(V,t)$ were periodic, the asymptotic behavior of $n_H(V,t)$, $t \to \infty$, also oscillated periodically (*43, 44*). We waited 300 ms before measuring each photoinduced DC current data point, which satisfied the asymptotic conditions for this molecular system (Fig. S4E to Fig. S4H).

The H and L state conductance (σ_H and σ_L) are taken to be constant and insensitive to light irradiation. Therefore, the tunneling current can be expressed by the equation:

$$I(V,t) = (n_H(V,t)\sigma_H + n_L(V,t)\sigma_L)V = n_H(V,t)\Delta\sigma V + \sigma_L V; \Delta\sigma = \sigma_H - \sigma_L \quad (S10)$$

With Eq. S10, we calculated the difference in current between two steady conditions (bright and dark):

$$I_{sub}(V) = I_{bright}(V) - I_{dark}(V) = \Delta n_{H,\infty}(V)\Delta\sigma V; \Delta n_{H,\infty} = n_{H,\infty,bright} - n_{H,\infty,dark} \quad (S11)$$

The lock-in amplifier setup measured the first harmonic component at the laser chopping frequency, $\frac{\omega}{2\pi}$. Using Eq. S10, we calculated the dynamic photoinduced DC current, $I_{DC}(V)$, by extracting the first harmonic Fourier series coefficient. We assumed the following form for the Fourier series:

$$I(V,t) = I_0(V) + I_\omega(V)\sin\big(\omega t + \theta_0(V)\big) + \cdots (higher\ harmonics) \quad (S12)$$

θ_0 is the phase between laser chopping and photoinduced DC current signal (Fig. S5). θ_0 is zero if n_H responds instantly to the laser modulation. For pyrrolidine at low bias voltage, θ_0 had a finite value because of the exponential response (Eq. S6). The observation of out of phase $I_{DC}(V)$ signal verified it. $I_\omega(V)\cos(\theta_0)$ and $I_\omega(V)\sin(\theta_0)$ corresponded to the in-phase and out-of-phase $I_{DC}(V)$.

In practice, the phase delay between laser chopping and lock-in reference also needed to be considered. However, we did not have a proper way to determine it. At high bias, we knew θ_0 is zero, but I_{DC} signal was too small to enable an accurate determination. For middle bias, I_{DC} signal was large, but θ_0 was difficult to determine. θ_0 and phase lag due to the electronics involved in the signal transmission were always coupled with each other (Fig. S5). We simply chose the phase that optimized the in-phase signal in the experiment. The phase between laser chopping and lock-in reference was a constant, which did not affect the identification of the I_{DC} signal.

Here we summarize steps used for calculating $I_{DC}(V)$ and $I_{sub}(V)$:

1. Obtain the fitting results for the transition rates.
   a. Fit the measured dark transition rate to inelastic tunneling model (use Eq. S1 and Eq. S2).
   b. Determine the photon contribution to transition rates by averaging the measured $W_{L/H\to H/L,bright}(V) - W_{L/H\to H/L,dark}(V)$.
2. Calculate $I_{sub}(V)$.
   a. Use the fitted transition rates (obtained from step 1) to calculate the equilibrium occupation values for bright and dark conditions ($n_{H,\infty,bright}$ and $n_{H,\infty,dark}$) from Eq. S8.
   b. Use the results obtained from step 2a to calculate $I_{sub}(V)$ from Eq. S11.
3. Calculate $I_{DC}(V)$.
   a. Obtain mathematical expression for $W_{L/H\to H/L}(V,t)$ under chopping laser condition (use Eq. S9 and fitting results obtained from step 1).
   b. Numerically solve $n_H(V,t)$ (use Eq. S6).
   c. Calculate $I(V,t)$ (use Eq. S10).
   d. Extract the first harmonic Fourier series coefficient for the current (use Eq. S12).
   e. Adjust the reference phase, so that the results from the calculation resemble the measurement.

Frequency-dependent $I_{DC}(V)$

Figure S6A and S6B show $I_{DC}(V)$ measured with different laser chopping frequencies. We found the maximum signal intensity to decrease as the chopping frequency increased (Fig. S6C). We attribute such effect to the limited dynamic response of the molecule. Among the four data points, only the 961 Hz measurement slightly falls outside the bandwidth of preamplifier

and could cause a small amount of signal reduction. However, the decreasing trend is evident even without this data point. A more accurate molecular frequency response requires calibrating the preamplifier frequency response.

The normalized in-phase $I_{DC}(V)$ spectra (Fig. S6A) showed subtle lineshape differences, but the peak intensity for out-of-phase $I_{DC}(V)$ increased with increasing chopping frequency (Fig. S5B). Such effects suggest that a finite molecular response can lead to a phase shift in the photoinduced DC current signal.

Current-dependent $I_{DC}(V)$

We observed three major variations in $I_{DC}(V)$ with increasing tunneling current setpoint (Fig. S7): an increase in the maximum signal, a red shift in the peak position, and a change in the lineshape.

With increasing tunneling current, the transition rates increase at all biases where inelastic tunneling electrons can cause two-level switching of the molecule. According to Eq. S7, the increased transition rates can increase the n_H decay rate within each half of the modulation period, leading to larger oscillation amplitude of tunneling current, especially for low bias region. Therefore, the I_{DC} increases, and the peak appears red-shifted due to enhanced signal at the lower magnitude of the sample bias. In addition, the increased σ_H and σ_L at larger tunneling current setpoints also contribute to the increased photoinduced DC current signal according to Eq. S10.

The current-dependent $I_{DC}(V)$ and bias dependence suggest that the dynamic response of pyrrolidine to photon irradiation can be controlled by electron flux and energy for tuning the single-molecule transitions between two states.

Power-dependent and Polarization-dependent $I_{DC}(V)$

Any laser characteristics that affect transition rates can potentially modify photoinduced DC current signal in two ways: changing the fractional occupation or population of the two states or changing the dynamic response. When $W_{L/H\to H/L,ph}$ is changed, the fractional occupation difference between dark and bright conditions is altered (Eq. S8), which can affect I_{sub} and I_{DC}. At the same time such change also affects $W_{L\to H} + W_{H\to L}$ and modifies the molecule's dynamic response at a fixed chopping frequency and I_{DC}.

Light induced surface plasmon can generate hot electrons that excite the molecular two-state transition. While photo-generated hot electrons primarily induce molecular transitions, excitation of molecular vibrations by Raman scattering also can contribute (*37*). Surface plasmons are sensitive to laser power and polarization. Therefore, both laser power and polarization variation can change $W_{L/H\to H/L,ph}$ and $I_{DC}(V)$ (Fig. S8).

$I_{DC}(V)$ of a Non-switching Molecule

Another possible origin of photoinduced DC current is the I-V nonlinearity of the tunneling junction (*7–9*). In this case the light acts as a fast-oscillating electric field and develops an AC voltage across the tunnel junction. The corresponding $I_{DC}(V)$ should share similar shape

with the $\frac{d^2I}{dV^2}$. To investigate this, we measured the IETS and $I_{DC}(V)$ of a carbon monoxide (CO) molecule adsorbed on Cu(001). Unlike pyrrolidine molecules, CO molecule does not undergo conformational switching or exhibit two-level properties. Therefore, CO is not expected to yield photoinduced DC current signal with the mechanism demonstrated for pyrrolidine. As shown in Fig. S9, CO molecule exhibited a large I-V nonlinearity in the measured bias region, however, I_{DC} signal was not observed after proper Z-compensation. We attribute the measured results to the small laser power used in the measurement, and the AC voltage across the tunnel junction was negligible. However, a high laser power destabilized the tunneling junction at cryogenic temperatures, leading to instability of the tunneling gap. We expect a short-pulsed laser would provide a better opportunity to observe photoinduced DC current generated by I-V nonlinearity and sufficient AC voltage across the tunnel junction. Because the short-pulsed laser can have a strong peak field but a low average power to produce the required AC voltage across the tunnel junction. Together with I-V nonlinearity, photoinduced DC current can be produced without detrimental thermal perturbation of the tunnel junction.

Photon Excitation Mechanisms

To investigate the excitation mechanisms of pyrrolidine conformational switching, we measured the power dependence of the transition rate at fixed bias (Fig. S10). We found that the transition rates in both directions depend linearly on the laser power P, $W_{L\rightarrow H} = 503.1P + 8.9$ and $W_{H\rightarrow L} = 182.1P + 15.4$. Such linear relation suggests excitation mechanisms dominated by a single photon process. We further define the slope of the linear relation as the efficiency of light assisted switching. We observed the efficiency differed significantly with the tip apex geometry, indicating mechanisms involving the tip enhanced surface plasmons in the STM junction. The induction of conformational switching of pyrrolidine molecule had been associated with the excitation of its vibrational modes (*38–41*).

The localized junction plasmons decay and generate a broad energy spectrum of hot electrons that can excite pyrrolidine vibrational modes and directly transitions between the two levels in a double-well potential of the molecule in the junction. These excitations facilitate the switching process. The pyrrolidine transitions respond rapidly to the light illumination, unlike the slower response expected for local heating assisted molecular switching. Thus, the conformational transition is induced by a nonthermal effect and unlikely due to laser induced heating. The linear laser power dependence in Fig. S10 is also consistent with a nonthermal mechanism.

Figures:

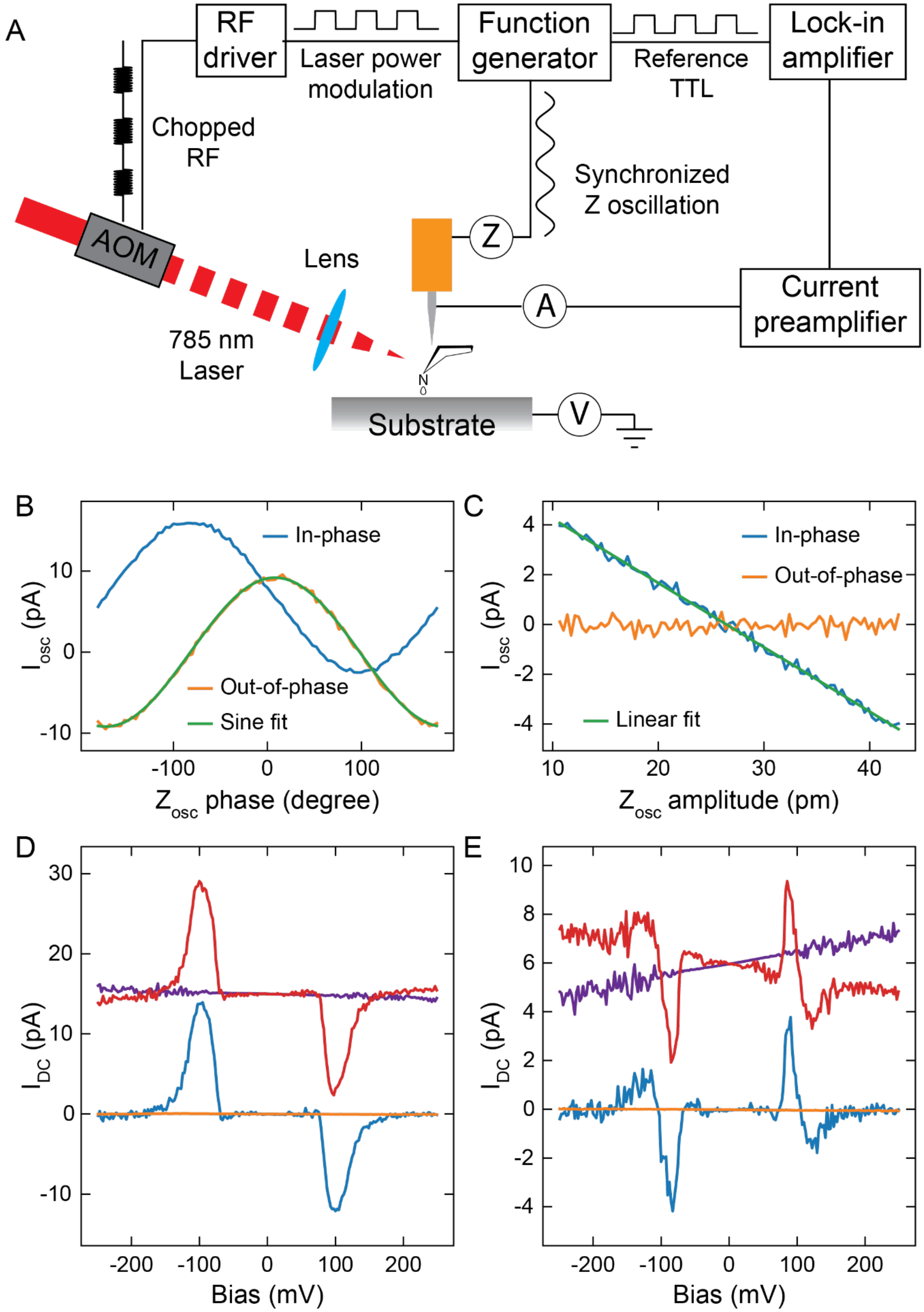

A
RF driver
Laser power modulation
Function generator
Reference TTL
Lock-in amplifier
Chopped RF
Synchronized Z oscillation
AOM
Lens
Z
A
785 nm Laser
Current preamplifier
Substrate
V
B
In-phase
Out-of-phase
Sine fit
I_{osc} (pA)
Z_{osc} phase (degree)
C
In-phase
Out-of-phase
Linear fit
I_{osc} (pA)
Z_{osc} amplitude (pm)
D
I_{DC} (pA)
Bias (mV)
E
I_{DC} (pA)
Bias (mV)

Fig. S1. Z compensation method and validation. (A) Schematic diagram of STM photoinduced DC current measurement. The function generator generates two synchronized signals: laser power modulation and Z oscillation. The synchronized Z oscillation drives the STM tip motion to compensate for the thermal expansion at the tunneling junction induced by laser chopping. (B) The tunneling current oscillation amplitude as a function of Z oscillation phase was measured by lock-in detection. A 327 Hz square-wave chopped laser with peak power of 2.98 mW was focused into the tunneling junction. The applied Z oscillation was synchronized to the laser power modulation with an amplitude of 35.6 pm. The fitted sine function for out-of-phase signal (green curve) is $18.2 \sin(98.14 - \theta_{zosc})$. (C) The tunneling current oscillation amplitude as a function of Z oscillation amplitude for the same laser irradiation as (B). The Z oscillation phase was fixed at its optimal value 98.14 degrees. The linear fit function is $-0.259\ (A_{zosc} - 26.344)$. Both (B) and (C) were measured when the tip was positioned over the substrate with set point -50 mV/1 nA and feedback loop open. (D) and (E) are in-phase and out-of-phase photoinduced DC current measurements. The red and blue $I_{DC}(V)$ curves were taken over pyrrolidine without and with Z compensation. The green $I_{DC}(V)$ curves were taken over pyrrolidine with only Z oscillation but no laser radiation. The orange curves were measured over metal substrate with both chopped laser irradiation and Z compensation.

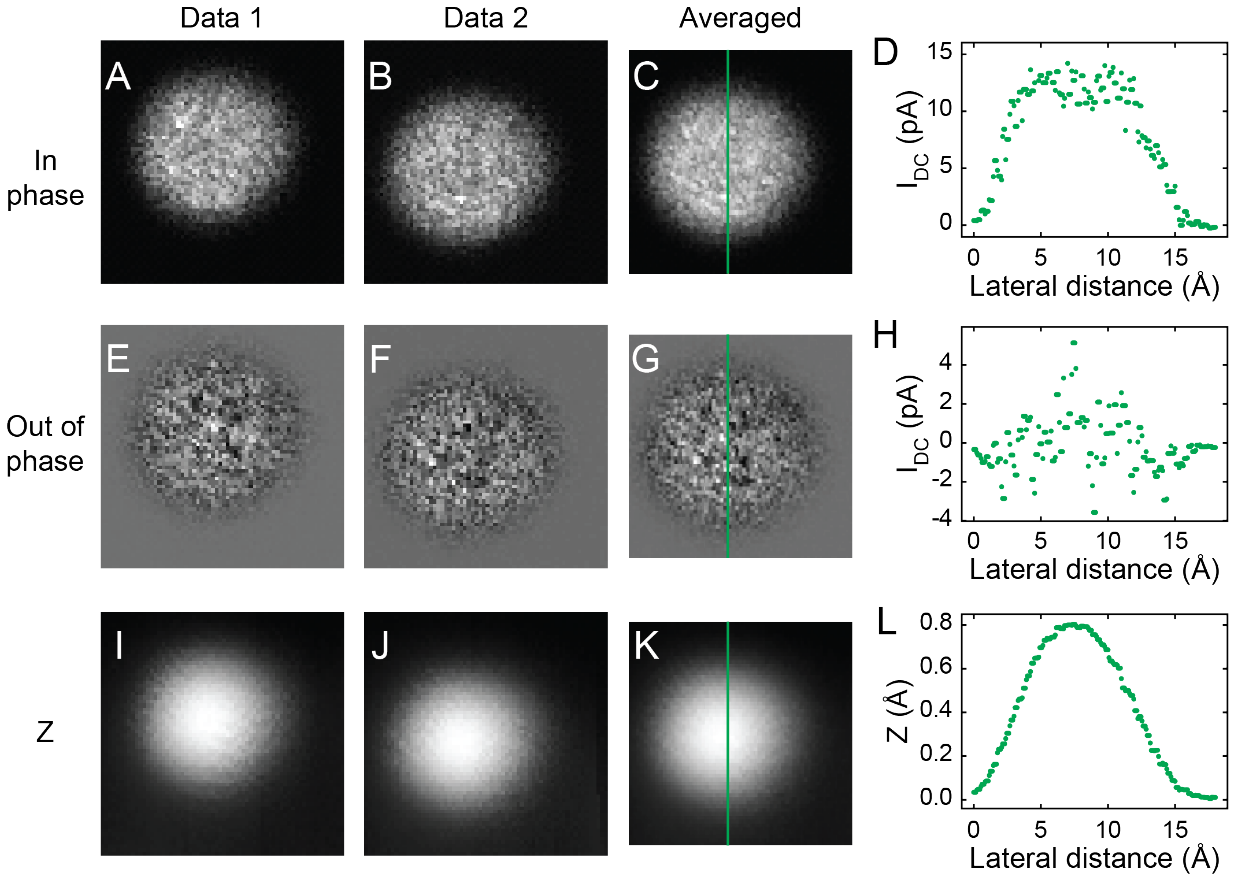

Fig. S2. Photoinduced DC current imaging and averaging. (A), (E), and (I) are simultaneous in-phase I_{DC} imaging, out-of-phase I_{DC} imaging, and topography with image size of 64x64 pixels. For each pixel, we performed the following measurement sequence: (1) maintained tunneling gap with feedback set point -250 mV/0.75 nA; (2) measured the topography signal; (3) opened the feedback loop; (4) set bias to -100 mV; (5) waited 300 ms and measured photoinduced DC current signal. (B), (F), and (J) are the second data set. (C), (G) and (K) are images from averaging data set 1 and data set 2. (D), (H) and (L) are the line cuts of the corresponding averaged images as indicated by the green lines in (C), (G), and (K).

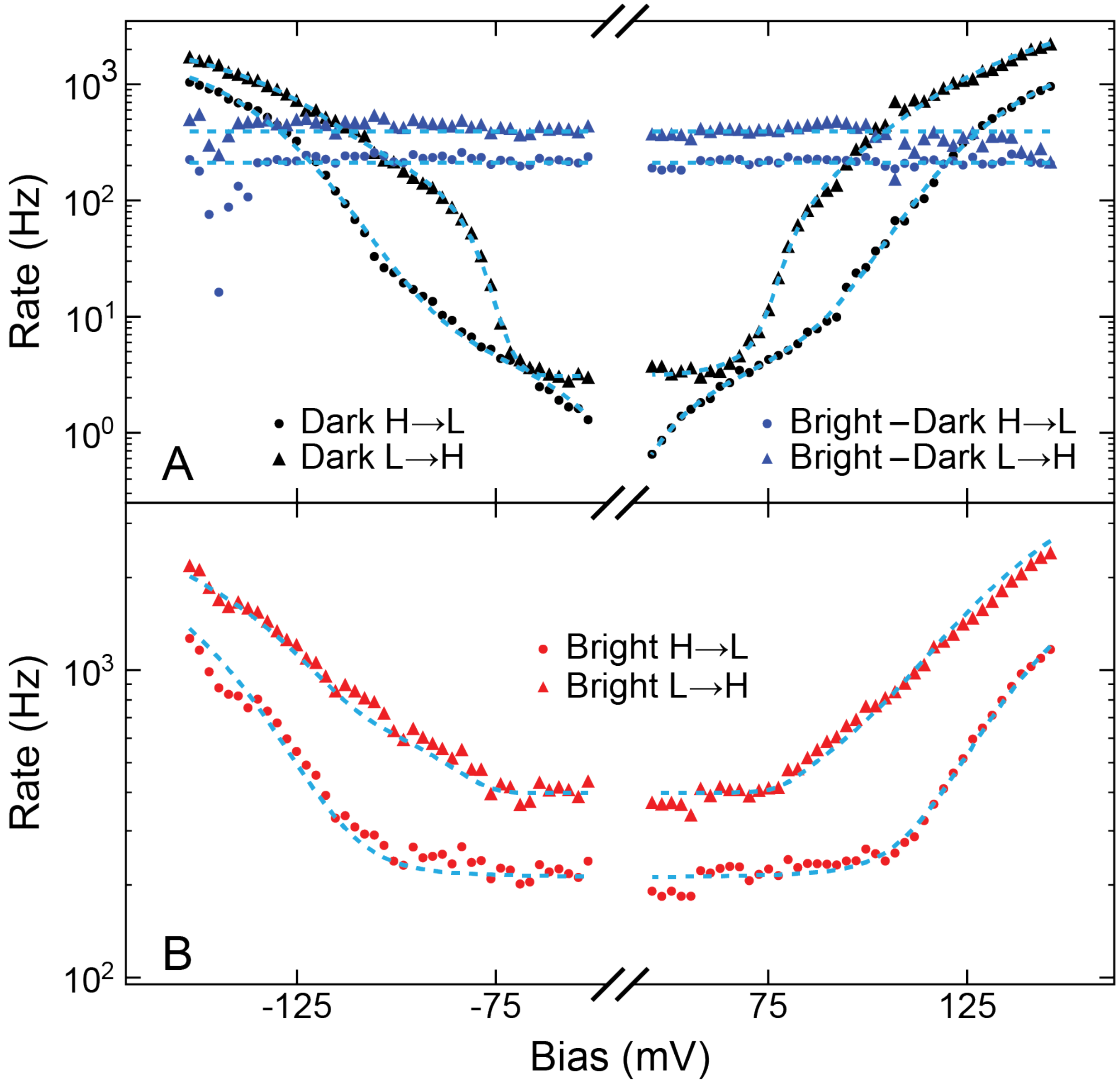

Fig. S3. Pyrrolidine transition rate as a function of sample bias. (A) and (B) The dark and bright transition rates as a function of sample bias. The green dashed lines are the fitted data. The fitting of the dark transition rate is based on switching induced by inelastic tunneling electrons. The subtractions of dark from bright rates are fitted to constants, which represent the photon-induced transition rates. The fitting of bright transition rates in (B) is the sum of the dark and the fitted photon-induced constant transition rates. The dark and bright transition rates were extracted from multiple long current traces at each bias with the same tunneling gap determined by the set point -250 mV/100 pA.

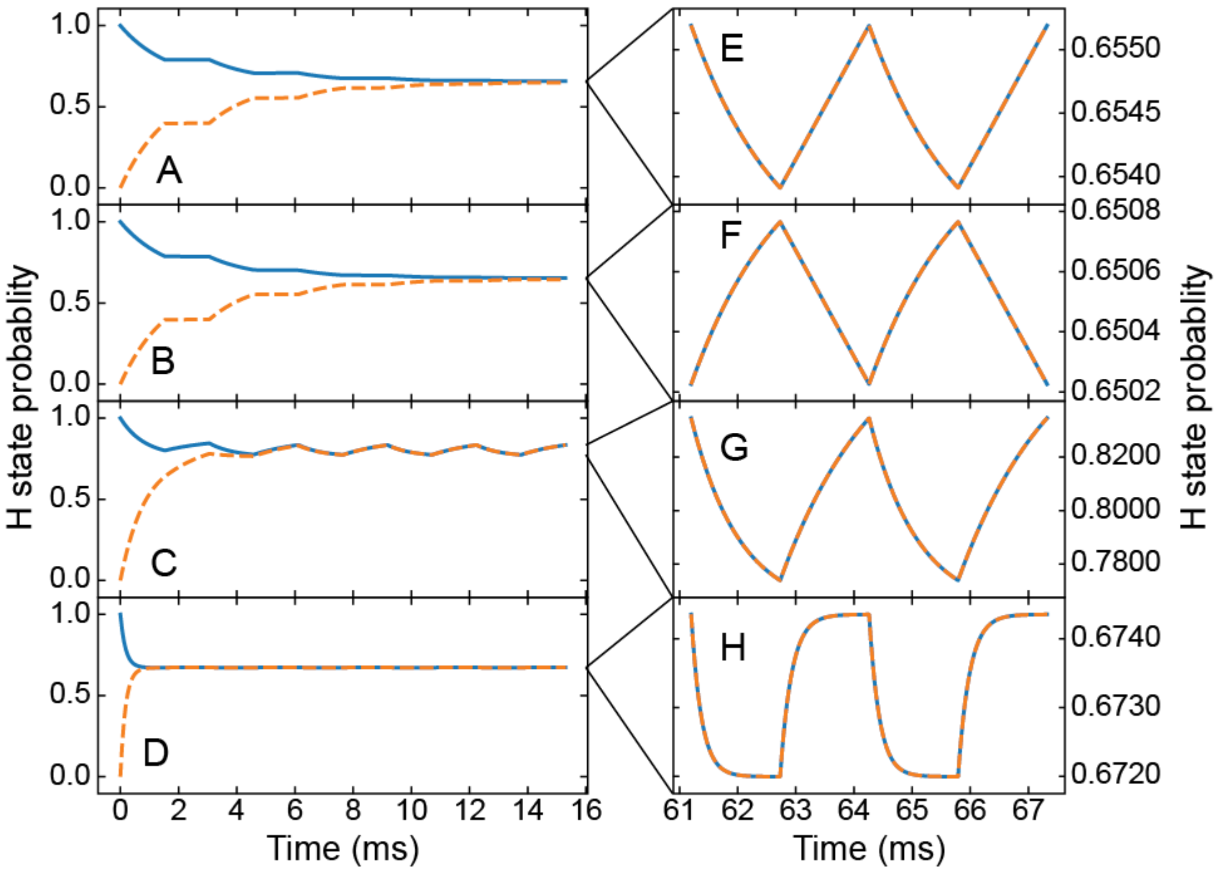

Fig. S4. Calculated time evolution of H state occupation probability (population) for pyrrolidine. (A)-(D) Simulated time evolution of H state occupation probability for pyrrolidine within the first few laser power modulation cycles for four different biases (40, 67, 109, and 200 mV, the same as in Fig. 5C). The blue and orange curves correspond to the molecule starting in the H state and L state, respectively. (E)-(H) The H state occupation probability after the first 20 laser power modulation cycles for each bias corresponding to (A)-(D), sufficient time for the H state probability to settle.

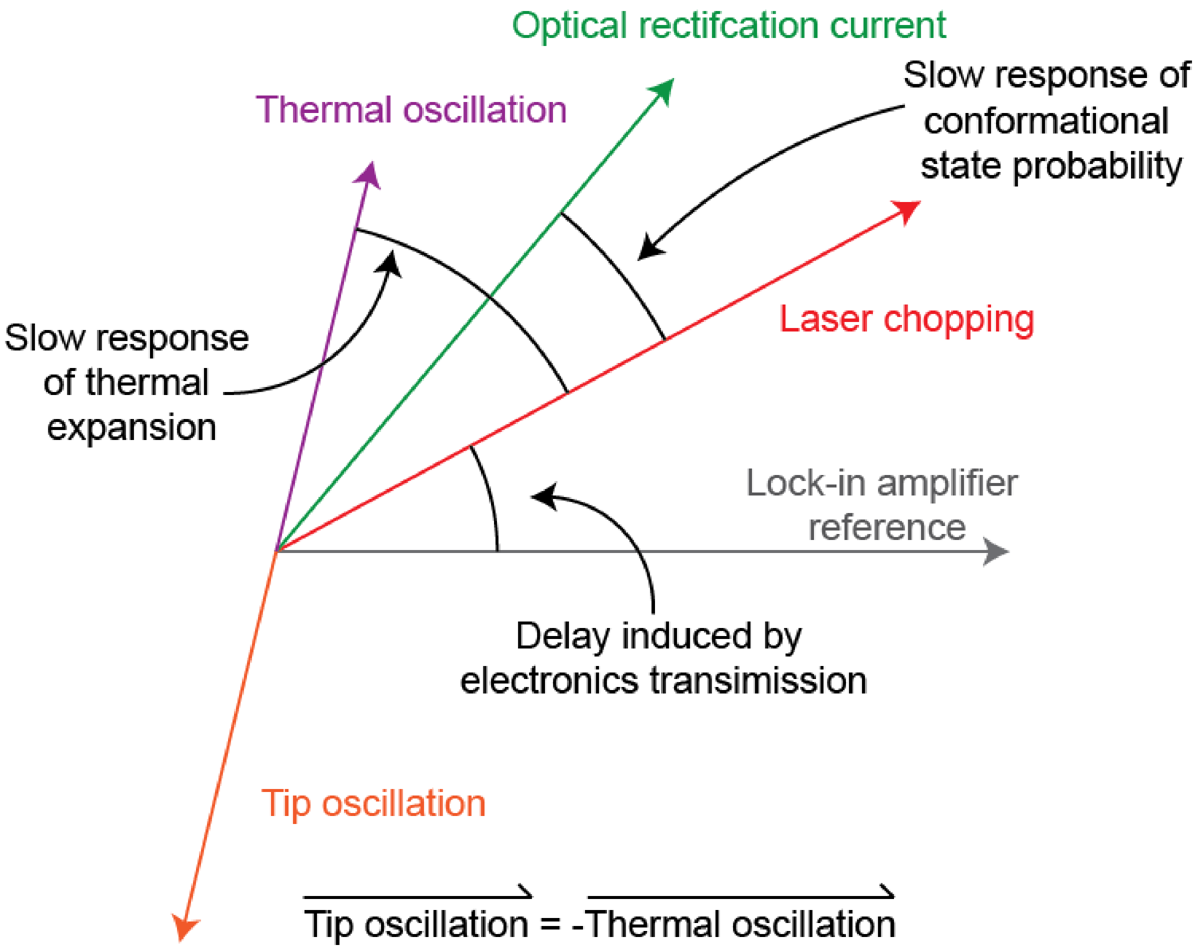

Fig. S5. Vector diagram of different signals and their phase relations.

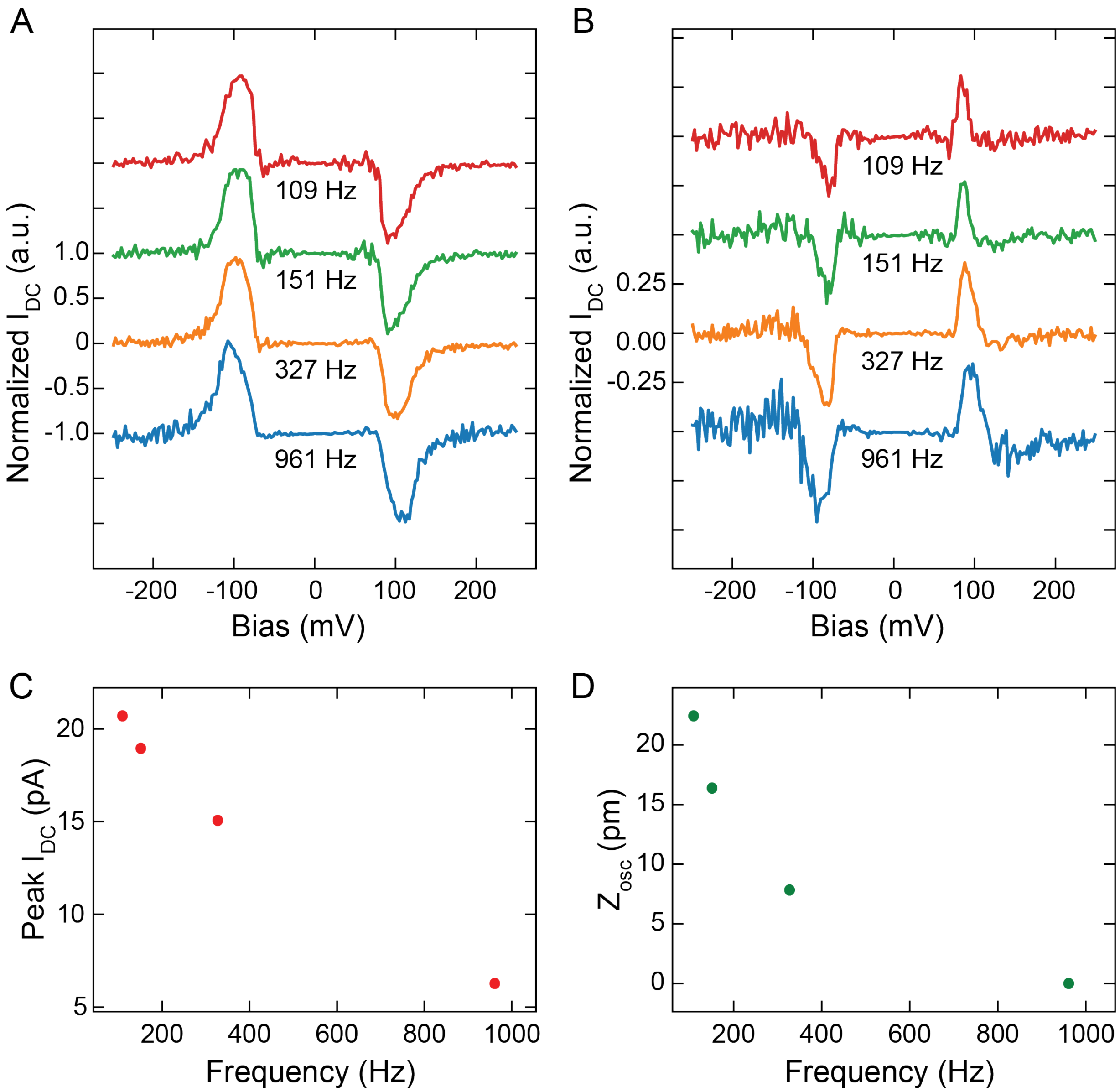

Fig. S6. $I_{DC}(V)$ and Z_{osc} at different modulation frequencies of the laser intensity. (A) and (B) are the in-phase and out-of-phase $I_{DC}(V)$ measured over the center of a pyrrolidine for different laser chopping frequencies. Each spectrum displayed is normalized by its maximum signal magnitude. Laser peak power of 0.95 mW and tunneling gap set by -250 mV/0.75 nA were used for all measurements. (C) The maximum intensity as a function of the laser chopping frequency extracted from the in-phase measurement in (A). (D) The optimal Z oscillation amplitude used in thermal compensation for each laser chopping frequency.

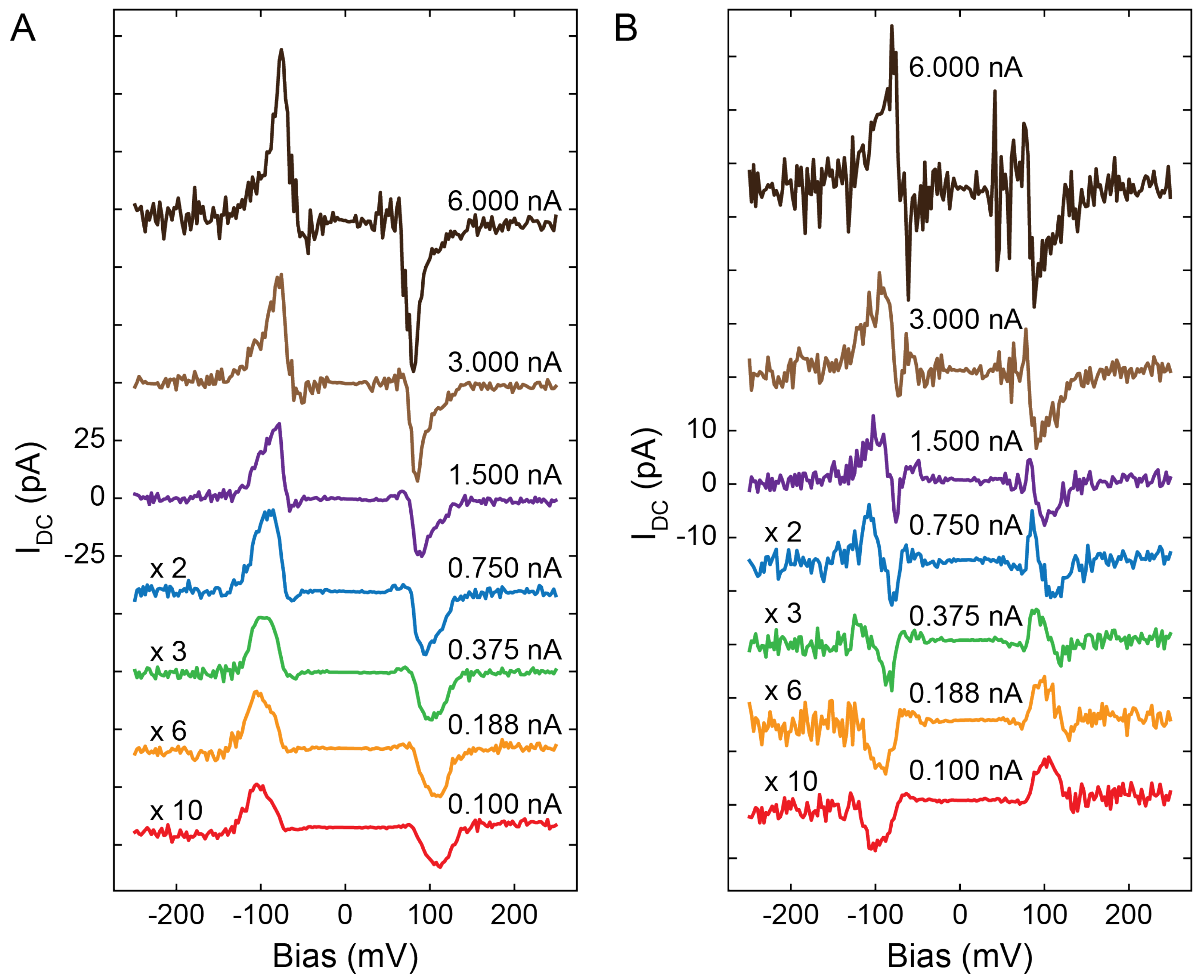

Fig. S7. $I_{DC}(V)$ for different tunneling current set point. (A) and (B) are the in-phase and out-of-phase $I_{DC}(V)$ measured over center of a pyrrolidine for different set point current with the set point bias fixed at -250 mV. A 327 Hz square wave-chopped laser radiation with a 0.95 mW peak power was used for all measurements. Spectra displayed are scaled as shown and offset for clarity.

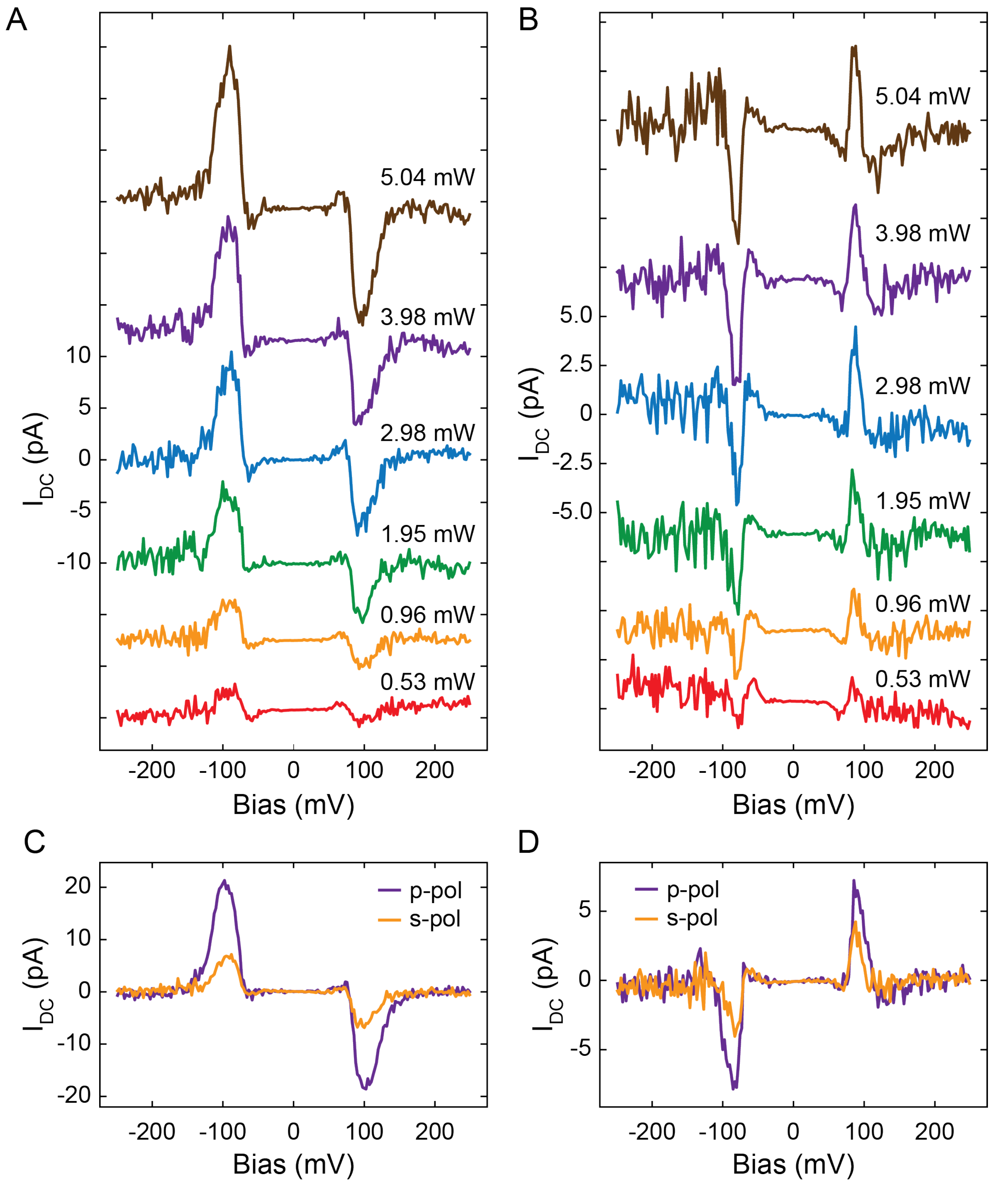

Fig. S8. $I_{DC}(V)$ for different power and polarization of laser light. (A) and (B) are the in-phase and out-of-phase $I_{DC}(V)$ measured over center of a pyrrolidine for different peak laser powers. Spectra displayed are offset for clarity. (C) and (D) are the in-phase and out-of-phase $I_{DC}(V)$ for two polarizations and a peak laser power of 0.48 mW; s-polarization is perpendicular to tip axis and angle of incidence is 45°. All $I_{DC}(V)$ were measured with tunneling gap set point of -250 mV/0.75 nA. The power series and polarization dependence of $I_{DC}(V)$ were measured with two different tips.

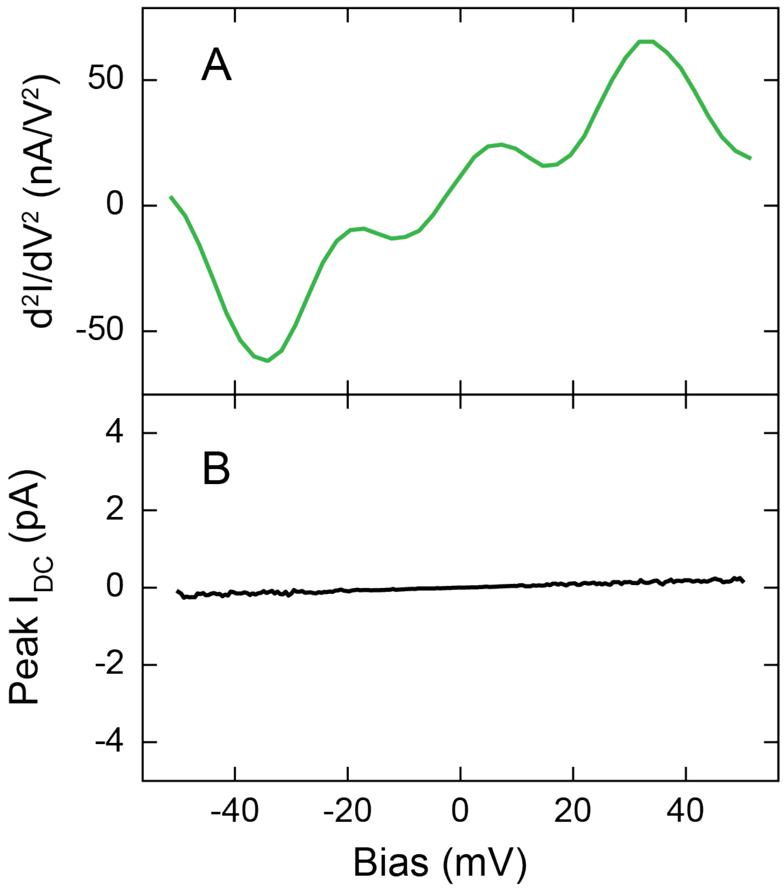

Fig. S9. IETS and $I_{DC}(V)$ for a carbon monoxide (CO) molecule adsorbed on Cu(001). (A) IETS and (B) $I_{DC}(V)$ for a CO molecule. The AC bias voltage modulation used for IETS measurement was 273 Hz, 3 mV_{rms}. Both spectra were measured with tunneling gap set point of -50 mV/1 nA. The amplitudes of laser power modulation and Z-oscillation were 1.2 mW and 10.1 pm, respectively. The phase shift for Z-oscillation was 83.94 degrees.

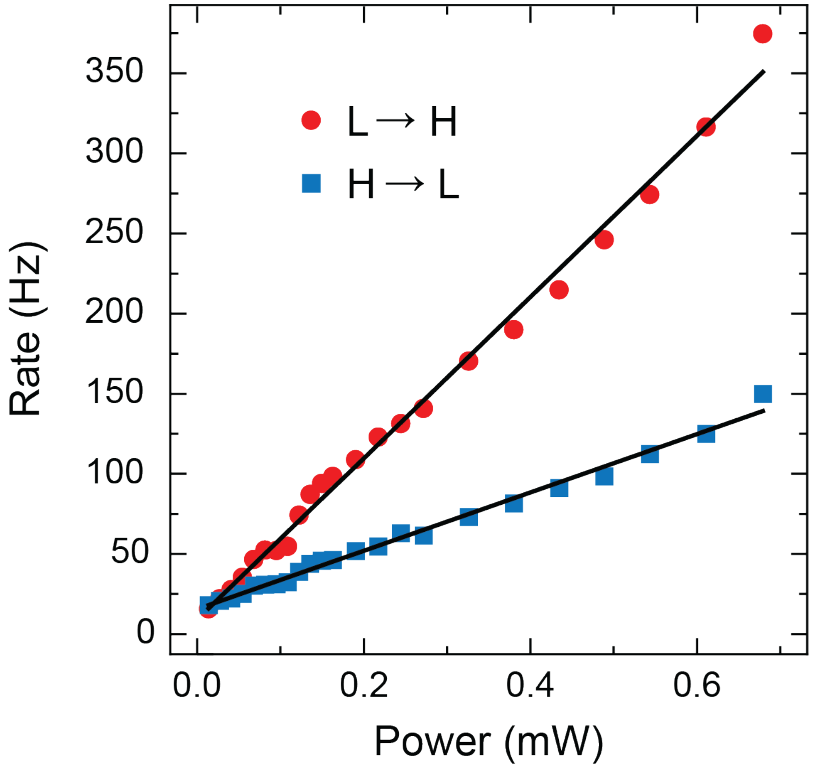

Fig. S10. Power dependence of pyrrolidine transition rate. Pyrrolidine low (L)-to-high (H) and high (H)-to-low (L) transition rates as a function of power for 1544 nm CW laser light, measured at 50 mV sample bias and with feedback turned off at set point -500 mV and 1 nA. Black lines are the linear fit of the corresponding data. Similar linear dependence is expected for 785 nm CW laser light used to take all other data in the manuscript and the Supplementary Materials, considering resonant excitation does not occur at these two wavelengths.

Tables:

Table S1: Fitting parameters for dark transition rates.

Transition	$const$ (Hz)	Vibrational Mode	$\hbar\Omega_{\nu}$ (meV)	$k_B T_{eff}$ (meV)	K_{ν} (Hz)	m_{ν}
$L \rightarrow H$	3.1	In-plane ring deformation	77.9	1.5	9.0	1
		Ring breathing	111.0	5.9	46.3	1
$H \rightarrow L$	0.31	Out-of-plane ring deformation	43.3	1.7	0.1	1
		Ring breathing	120.4	5.6	37.9	1

Table S2: Photon contribution used for fitting bright transition rates.

Transition	$W_{L/H \to H/L,ph}$ (Hz)
$L \to H$	395.5
$H \to L$	211.4
